# Automated Detection of Parkinson’s Disease Based on Multiple Types of Sustained Phonations Using Linear Discriminant Analysis and Genetically Optimized Neural Network

**DOI:** 10.1109/JTEHM.2019.2940900

**Published:** 2019-10-07

**Authors:** Liaqat Ali, Ce Zhu, Zhonghao Zhang, Yipeng Liu

**Affiliations:** School of Information and Communication EngineeringUniversity of Electronic Science and Technology of China (UESTC) Chengdu 611731 China

**Keywords:** Dimensionality reduction, genetic algorithm, hyper-parameter optimization, linear discriminant analysis, Parkinson’s disease, deep neural network

## Abstract

Objective: Parkinson’s disease (PD) is a serious neurodegenerative disorder. It is reported that most of PD patients have voice impairments. But these voice impairments are not perceptible to common listeners. Therefore, different machine learning methods have been developed for automated PD detection. However, these methods either lack generalization and clinically significant classification performance or face the problem of subject overlap. Methods: To overcome the problems discussed above, we attempt to develop a hybrid intelligent system that can automatically perform acoustic analysis of voice signals in order to detect PD. The proposed intelligent system uses linear discriminant analysis (LDA) for dimensionality reduction and genetic algorithm (GA) for hyperparameters optimization of neural network (NN) which is used as a predictive model. Moreover, to avoid subject overlap, we use leave one subject out (LOSO) validation. Results: The proposed method namely LDA-NN-GA is evaluated in numerical experiments on multiple types of sustained phonations data in terms of accuracy, sensitivity, specificity, and Matthew correlation coefficient. It achieves classification accuracy of 95% on training database and 100% on testing database using all the extracted features. However, as the dataset is imbalanced in terms of gender, thus, to obtain unbiased results, we eliminated the gender dependent features and obtained accuracy of 80% for training database and 82.14% for testing database, which seems to be more unbiased results. Conclusion: Compared with the previous machine learning methods, the proposed LDA-NN-GA method shows better performance and lower complexity. Clinical Impact: The experimental results suggest that the proposed automated diagnostic system has the potential to classify PD patients from healthy subjects. Additionally, in future the proposed method can also be exploited for prodromal and differential diagnosis, which are considered challenging tasks.

## Graphical abstract

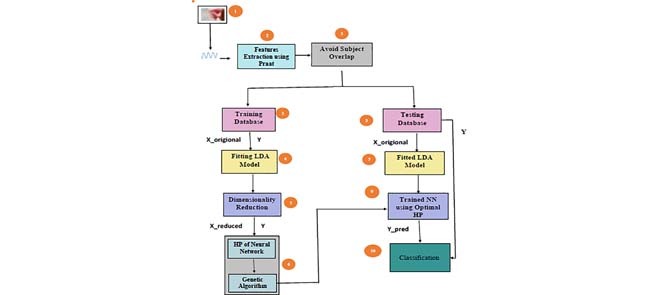

## I. Introduction

After Alzheimer’s disease (AD), Parkinson’s disease (PD) has become the second most common neurological syndrome of the central nervous system [1]. PD is a serious neurodegenerative disease and targets elder people mostly after the age of 60 years [2]. Patients with PD (PWP) are generally characterized by bradykinesia (slowness of movement), dysphonia (voice impairments), rigidity, tremor, and poor balance [3]–[6]. Vocal impairments are considered to be one of the earliest symptoms of the disease [7]. Hence, diagnosis of PD based on acoustic analysis of subjects is considered to be an early detection of the disease [8]. These factors motivated the use of voice recording based data for the PD diagnosis [9]–[14]. At early stages, PD patients have voice impairments that might not be perceptible to listeners. However, these impairments can be detected by performing acoustic analysis [15]. In order to detect these abnormalities in the voice, we develop an automated system that has the capability to discriminate voice of PD patients from that of healthy subjects.

Patients having rapid eye movement sleep disorder (RBD) are at substantial risk (*i.e*. having high probability) for developing PD. It has been shown that slight speech impairment may be a sensitive marker of early degeneration *i.e*., prodromal neurodegeneration [16], [17]. Hence, the proposed LDA-GA-NN system (binary classification) can also be applied in future studies for the detection of prodromal neurodegeneration. Additionally, recent research shows speech-based differential diagnosis of PD. Rusz *et al.* carried out a comprehensive study to show that motor speech function can be used to differentiate between PD, Parkinsonian variant of multiple system atrophy (MSA) *i.e*. MSA-P and cerebellar variant of MSA *i.e*. MSA-C [18]. The proposed learning system can also be exploited for such a differential diagnosis by modelling the problem as multi-class classification problem. The only difference will be the size of the obtained feature vector *i.e*. in case of LDA, the reduced feature vector size is equal to $C-1$ where $C$ denotes the number of classes in the classification problem. Hence, for differential diagnosis with four classes *i.e*., PD, MSA-C, MSA-P and healthy control, the proposed method will result in a 3-dimensional feature vector. In such a case, a search algorithm will have to be exploited to search out which of the three coordinates of the reduced feature vector to use.

Recent research shows development of different diagnostic systems based on machine learning and data mining approaches for the detection of PD to analyze hand written patterns, voice signals, physiological signals, wearable sensors for gait analysis, etc [6], [19]–[22], [22]–[26]. However, among these different analytical methods, speech recording based methods have drawn significant attention owing to the above discussed advantages of speech data for PD detection. Little *et al.* utilized replicated voice data from 31 subjects and achieved PD detection accuracy of 91.4% through support vector machine (SVM) [6]. Guruler *et al.* utilized complex-valued artificial neural network with k-means clustering based feature weighting method for the same dataset and achieved the highest classification accuracy of 99.52% [19]. However, the main problem in this dataset was its imbalance nature and most of the machine learning algorithms are very sensitive to imbalance classes. Additionally, most of the methods applied on this dataset utilized conventional k-fold cross validation which results in subject overlap. Hence, Sarkar *et al.* collected a balanced dataset by recording different types of speech and sustained phonation samples from 68 subjects [20].

Sarkar *et al.* collected and arranged the data into two databases, one was named training database and second testing database [20]. They utilized k-nearest neighbour model and SVM for classification purposes and obtained PD detection accuracy of 55%. To improve the PD detection accuracy, different researchers utilized different features selection methods [19], [25], [27]–[33]. For example, Naranjo *et al.* proposed a two stage variable selection and classification method for diagnosis of PD [27]. Canturk *et al.* attempted to enhance the PD prediction accuracy by exploring different feature selection and classification algorithms and could achieve accuracy of 57.5% under Leave one subject out (LOSO) cross validation (CV) [29]. Li *et al.* obtained accuracy of 82.5% by utilizing a hybrid feature learning approach and SVM model [30]. Recently, Benba *et al.* extracted features in cepstral domain using mel-frequency cepstral coefficients (MFCCs) [1]. They selected different features iteratively and applied the subset of the selected features to SVM for classification. They achieved classification accuracy of 82.5% for LOSO CV. Benba *et al.* in [31] obtained 87.5% accuracy by using only vowel samples and a subset of human factor cepstral coefficients (HFCCs). Most recently, Vasquez-Correa used deep learning for quantification of transition between voicing in PD compared to healthy subjects and validated their experiment using an independent dataset in three different languages [34].

In literature, different researchers have developed LDA driven machine learning models and achieved state-of-the-art performance on different health informatics problems. For example, Abdulkadir Sengur developed an expert system named LDA-ANFIS which used LDA for dimensionality reduction and adaptive neuro-fuzzy inference system (ANFIS) for classification [35]. The expert system achieved 95.9% sensitivity and 94% specificity rate for heart valve disease detection. Dogantekin *et al.* also checked the feasibility of LDA-ANFIS for the classification of hepatitis disease and achieved accuracy of 94.16% [36]. Subasi and Gursoy developed a cascaded system using LDA for dimensionality reduction and SVM for classification in order to automate detection of epilepsy [37]. Calisir and Dogantekin developed LDA-WSVM which used LDA for dimensionality reduction and wavelet based support vector machine for the automatic classification of diabetes [38].

**FIGURE 1.**
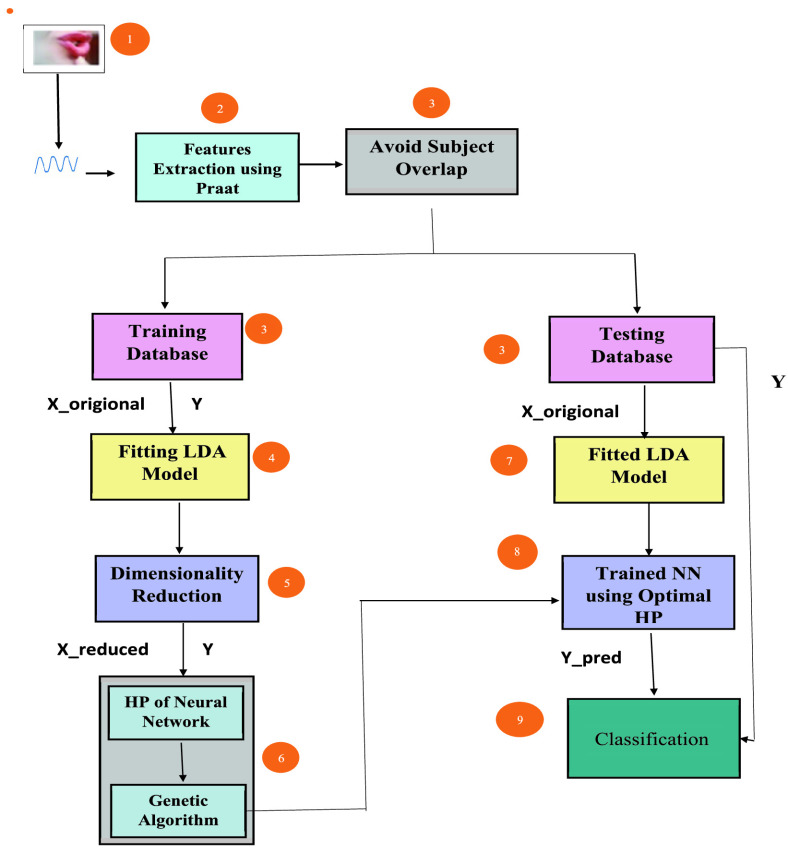
Flow chart of the proposed diagnostic system. HP: Hyperparameters, NN: Neural Network, X_origional: Origional Feature Vector, X_reduced: Reduced Feature Vector, Y: True Label, Y_pred: Predicted Label.

Motivated by the development of different automated expert systems based on LDA and other predictive models, we also attempted to develop an automated system for the effective detection of PD. Our proposed automated system overcomes the problems present in the previously proposed methods. For example, the methods proposed in [1], [20] and [31] lack generalization and produce lower PD detection accuracy which has limited clinical significance. Similarly, the automated system developed in [25] has the problem of subject overlap caused by conventional k-fold validation scheme. Additionally, the multiple types of voice or phonation data is imbalanced in terms of gender. However, many of the features of the dataset are gender dependant. Hence, these approaches applied to multiple voice data have still left some challenges open. To overcome these challenges, we propose a hybrid intelligent system namely LDA-NN-GA that uses LDA for dimensionality reduction and GA for optimization of NN which is used for classification. Moreover, to obtain a model that could show better performance on training data and generalize to testing data, we repeat the optimization through GA for K-times in order to obtain K best models that are genetically optimized. Finally, we select that model which shows good performance on the training database and the testing database *i.e*. that has better generalization capabilities. Additionally, to avoid the impact of gender dependant features, we perform an additional experiment by simulating the proposed system on feature set without using the gender dependent features. The working of the proposed LDA-NN-GA is more clearly depicted in Fig. 1. In summary, the main contributions of the study can be summarized as:

1. A hybrid intelligent system, *i.e*., LDA-NN-GA, is developed and applied for the first time to the PD detection problem.
2. The proposed system achieves outstanding performance compared with the previous methods that used all the features of the multiple types of phonations data.
3. Additionally, the model constructed in this study has generalization capabilities which is the property that was not observed in the previously proposed models. Furthermore, the proposed method offers lower complexity.
4. In this paper, we highlight a potential problem in the multiple types of phonations data that has been extensively used in the past for PD detection. The data is imbalanced in terms of gender while the feature space contains many gender dependent features. Thus, in this paper, we performed an additional experiment by eliminating the gender dependant features.

The organization of the remaining paper is as follows; information about the dataset and proposed method are given in section 2. Discussion about validation schemes and evaluation metrics is given in section 3. Experimental results and comparative study are given in section 4 and 5, respectively. Section 6 discusses the limitations of the study. The last section is about conclusion.

## II. Materials and Methods

### A. Dataset Description

The dataset used in this study was collected by Sarkar *et al.* in [20] at the Department of Neurology in Cerrahpasa, Faculty of Medicine, Istanbul University. The database is distributed in two parts *i.e*., training database and testing database. The training database contains data of 20 PD patients and 20 healthy subjects. The PD patients of the training database are suffering from the disease from 0 to 6 years and having ages in the range 43 and 77 with mean of 64.86 and standard deviation of 8.97. While the ages of the healthy subjects are in the range 45 and 83 with mean of 62.55 and standard deviation of 10.79. From each subject, multiple types of speech samples were recorded including short sentences, words, sustained vowels and numbers. From each subject 26 samples were recorded. Thus, the training database contains $40\times 26=1040$ samples in total. A Trust MC-1500 microphone was used for speech recordings. The distance between a subject and microphone was set to 15 cm. From each sample, 26 linear and time frequency based dysphonic features were extracted using Praat software [39]. A detail of these 26 features is given in Table 2. The second independent database was named testing database. The PD patients of the testing database were suffering from the disease from 0 to 13 years and the ages of the subjects range between 39 and 79 with mean value of 62.67 and standard deviation of 10.96. The testing database contains 28 subjects and 168 samples as 6 samples were recorded from each subject including 3 phonations of sustained vowel “a” and three phonations of sustained vowel “o”. Thus, the testing database contains $28\times 6=168$ samples. The key component of the dataset is the training database as it is balanced in terms of healthy and PD groups and the PD patients also have same disease duration. That is why many of the previous studies used only the training database. Additionally, the authors of the dataset provided UPDRS III score for the training database only which have been reported in Table 1. UPDRS III *i.e*. motor UPDRS ranges from 0 to 108, where 0 represents symptom free and 108 represents severe motor impairments [40]. The motor UPDRS encompasses tasks such as speech, tremor, rigidity and facial expression [40]. In Table 2, the statistical parameters like mean, standard deviation and p value for each feature are also tabulated. The p value is calculated using Kruskal-Wallis test.

**TABLE 1:** Details of UPDRS III of PD patients

Subject ID	UPDRS	Subject ID	UPDRS
1	23	11	24
2	8	12	32
3	40	13	23
4	5	14	5
5	16	15	31
6	46	16	55
7	40	17	5
8	20	18	32
9	11	19	26
10	12	20	46

**TABLE 2:** Details of Extracted Features From Each Sample. Mean: Average of Each Feature Values, Std: Standard Deviation of Each Feature Values. $\mathrm{PD_{Tr}}$: PD Group of Training Database, $\mathrm{H_{Tr}}$: Healthy Group of Training Database, $\mathrm{PD_{Ts}}$: PD Group of Testing Database, M: Mean, S: Standard Deviation

Feature number	Features	$\mathrm {PD_{Tr}}$	$\mathrm {H_{Tr}}$	$\mathrm {PD_{Ts}}$	Statistical comparisons
	Description	(M ± S)	(M ± S)	(M ± S)	p value
1	Jitter (local)	0.79±0.66	0.77±0.75	0.64±0.82	0.0003
2	Jitter(local, absolute)	5.09e-05±4.29e-05	5.02e-05±5.20e-05	4.2e-05±5.5e-05	0.009
3	Jitter (rap)	0.42±0.41	0.392±0.466	0.33±0.44	0.007
4	Jitter (ppq5)	0.42±0.37	0.368±0.340	0.37±0.52	0.005
5	Jitter (ddp)	1.26±1.24	1.17±1.39	1.006±1.346	0.007
6	Shimmer (local)	7.18±3.38	6.85±5.48	5.162±3.060	0.006
7	Shimmer (local, dB)	0.681±0.34	0.62±0.45	0.47±0.26	<0.001
8	Shimmer (apq3)	3.53±2.09	3.54±3.09	2.67±1.79	0.039
9	Shimmer (apq5)	4.34±2.50	4.360±4.06	3.04±1.67	0.007
10	Shimmer (apq11)	6.23±3.38	5.34±3.78	4.21±2.03	0.001
11	Shimmer (dda)	10.59±6.29	10.62±9.87	8.03±5.38	0.039
12	Autocorrelation	0.94±0.051	0.93±0.089	0.96±0.048	0.001
13	Noise-to-Harmonic	0.066±0.077	0.092±0.151	0.051±0.083	<0.001
14	Harmonic-to-Noise	16.31±5.01	17.44±5.89	19.32±5.53	0.013
15	Median pitch	166.1±44.64	166.7±46.41	164.62±46.37	0.968
16	Mean pitch	166.3±44.58	169.8±47.14	163.94±46.18	0.928
17	Standard deviation	6.63± 4.57	14.2±23.80	4.99±7.11	<0.001
18	Minimum pitch	149.11±45.76	147.8±45.37	151.5±49.15	0.872
19	Maximum pitch	181.9±48.99	227.5±108.9	173.39±48.83	0.050
20	Number of pulses	390.7±350.8	321.4±205.09	157.60±46.90	<0.001
21	Number of periods	389.3±350.6	318.3±205.04	155.88±47.16	<0.001
22	Mean Period	0.0064±0.001	0.0062±0.001	0.0066±0.0021	0.958
23	Standard Dev. Of period	0.00027±0.0001	0.00040±0.00042	0.00024±0.00034	<0.001
24	Fraction of locally unvoiced frames	0.956±1.90	3.384±6.37	0.767±2.359	<0.001
25	Number of voice breaks	0.175±0.49	0.25±0.53	0.226±0.870	0.229
26	Degree of voice breaks	0.073±0.207	0.961±3.37	0.65±2.29	0.234

### B. Problem Formulation and Proposed Method

The main objective of supervised learning using machine learning methods is to search out such a model or generate such a hypothesis *i.e*., fitting function that would generalize to testing data. Hence, we use training database for construction of a model and then test its generalization capabilities by applying it to testing database. To improve the detection accuracy, the most pertinent way is to use some feature selection algorithms or feature extraction methods. Feature extraction is the process of deriving new features from original features in order to enhance classifier efficiency [41], [42]. Many feature extraction techniques involve linear transformations of the original pattern vectors to new vectors of lower dimensionality. In this paper, we propose to use LDA model to meet this objective. LDA is a dimensionality reduction method that is used at the initial level of a predictive model which is used for patterns classification. The main job of LDA is to search such vector(s) in the vector space that provide better separation of the classes of the data. The class separability can be evaluated by projecting the original data points on to these vector(s). Hence, if the classes are overlapped for a given data points, LDA tries to better separate them by applying some transformation mechanism. To meet this objective, LDA exploits a rule known as Fisher ratio *i.e*., LDA tries to maximize the fisher ratio which is formulated as follows:

\begin{equation*} \dfrac {(\mu _{1}-\mu _{2})^{2}}{\sigma
              _{1}^{2} + \sigma _{2}^{2}}\tag{1}\end{equation*}

where $\sigma _{1}$ denotes the variance of the first class and $\sigma _{2}$ the variance of the second class while $(\mu _{1}-\mu _{2})$ represents difference between center points or means of the two classes or distributions. Thus, by maximizing the Fisher ratio, LDA tries to maximize the distance between the two classes *i.e*., it maximizes the scatter between the two classes *i.e*. $S_{B}$ while it tries to make the two classes as condense as possible by minimizing $\sigma _{1}^{2} + \sigma _{2}^{2}$ *i.e*., it tries to reduce the within class scatter *i.e*. $S_{W}$. Thus, the fisher ratio given in (1) can be written as follows:

\begin{equation*} \dfrac {S_{B}}{S_{W}}\tag{2}\end{equation*}

Thus, our objective is to maximize (2) by transforming our data to lower dimensional space. To meet this objective, we need a transformation matrix $w$. We can write $S_{B}$ as follows:

\begin{align*} S_{B}=&w^{T}S_{B}w \tag{3}\\
              S_{W}=&w^{T}S_{W}w\tag{4}\end{align*}

Hence, (2) becomes

\begin{equation*} \dfrac
              {w^{T}S_{B}w}{w^{T}S_{W}w}\tag{5}\end{equation*}

Finally, we need to find a transformation matrix $w$ that maximizes (5). LDA model evaluates the $w$ matrix by calculating the eigenvectors of $S_{W}^{-1}S_{B}$. Thus, LDA uses the transformation matrix to transform data having $p$ dimension into $k$ dimension where $k< =(C-1)$ where $C$ are the number of classes in the dataset. In case of binary classification (disease detection), $C=2$ *i.e*. patient or healthy class and hence $k=1$. During the transformation through $w$, LDA ensures maximization of (2).

LDA has two advantages. First, it improves the strength of the predictive model by transforming or projecting the original feature vectors into reduced vector space where the class separability is maximized. Second, it reduces the time complexity of the predictive model enormously. After the dimensionality reduction by LDA, the transformed data is applied to neural network for classification.

The performance of neural network is highly dependent on its configuration or hyperparameters setting [43]. Inappropriate hyperparameters will lead to mediocre performance as it will result in underfitting or overfitting the network. A given neural network $A$ has two attributes attached to it. One is parameters or weights denoted by $\beta $, and the other is hyperparameters denoted by $\lambda $. In this paper, we have used two important hyperparameters of the neural network for optimization *i.e*. the number of hidden layers in neural network and neurons in each hidden layer. Optimal values of parameters can be calculated by a learning algorithm from the given training data by optimizing a cost function. The cost function used in this paper is cross entropy which is the measure of error between predicted label values *i.e*. $\hat {y}$ by NN and corresponding actual values *i.e*. $y$ in the training samples. We name this optimization problem as NN parameters optimization. To find optimal values of hyperparameters, we optimize another objective function known as validation loss (e.g., misclassification rate) that the model achieves on validation data when trained on some training data from the same dataset. We name this optimization problem as NN hyper-parameters optimization problem. The two optimization problems of NN are formulated as follows.

For the given $n$ number of training examples, a NN learns a hypothesis function *i.e*. a fitting function $h_{\beta }(x)$ parametrized by NN parameters $\beta $ where $x$ denotes the input feature vector. The job of $h_{\beta }(x)$ is to predict label $\hat {y}$ for a given input feature vector $x$. The goal is to find those optimized values of parameters $\beta $ that minimize the objective function as:

\begin{equation*} C(\beta) = \frac {1}{n} \sum
              _{j=1}^{n}cost(h_\beta (x^{(j)}), y^{j})\tag{6}\end{equation*}

To solve the minimization of (6), we used Inverse Broyden-Fletcher-Goldfarb-Shann (IBFGS) and Adam algorithms. The optimization algorithms are implemented in scikit-learn library of Python programming language. After the optimization of the parameters of the neural network model for training data, testing data samples are applied to the trained neural network to evaluate its performance. This gives rise to a hyper-parameter optimization problem of the neural network which can be formulated for LOSO CV with $k$ subjects as follows.

\begin{equation*} l(k) = \frac {1}{k} \sum _{i=1}^{k}\mathcal
              {L}(A_{\lambda }, D^{i}_{train}, D^{i}_{valid})\tag{7}\end{equation*}

The function $\mathcal {L}$ maps a hyperparameter choice or setting $A_{\lambda }$ to average of the validation error or loss achieved by the network in different folds of LOSO CV. Thus, we need to search optimal hyper-parameters $\lambda $ of NN that can minimize the validation loss. To solve the minimization of (7) *i.e*. to search optimal $\lambda $, we use GA algorithm in this paper.

Recently, genetic optimization based methods have been widely utilized in different applications [44]–[48]. To design an effective neural network model, values of its hyperparameters have to be chosen carefully. As the hyperparameter settings determine the architecture of the network. Additionally, network architecture with excessive capacity will result in overfitting while network configuration with insufficient capacity will lead to underfitting problem. In the proposed LDA-NN-GA method, the NN hyperparameters are dynamically optimized by implementing GA evolutionary process. The optimal values of the NN’s hyperparameters are searched by GA with randomly generated initial population consisting of chromosomes. The values of the two important hyperparameters of NN *i.e*., number of layers $L$ and number of neurons in each hidden layer $H_{L}$ are directly coded in the chromosomes. To assess the performance of each chromosome, a fitness function is designed.

In this paper, we design the fitness function as the generalization performance achieved over LOSO CV so that the genetically searched optimal hyperparameters could give us highest prediction accuracy and generalization ability simultaneously. The proposed GA uses selection, crossover, and mutation operators to generate the offspring of the existing population. In the selection stage, two types of methodologies are usually used *i.e*. roulette wheel method and tournament selection. In this paper, we implemented tournament selection method. In the selection stage, those individuals *i.e*. chromosomes that are the fittest and survive to the next generation are placed in a matting pool for crossover and mutation operations. In the cross over stage, one or more randomly selected positions are assigned to the selected chromosomes that are to be crossed. To generate new population, the newly crossed chromosomes are combined with the rest of the chromosomes. At the mutation stage, the mutation operation determines whether a chromosome should be mutated in next generation or not based on a predefined probability known as mutation probability. After mutation, new population is generated and the same process is repeated for prescribed number of generations. Finally, the algorithm will return that hyperparameter setting that will offer minimum validation loss or maximum validation accuracy achieved over LOSO CV. After obtaining the optimized NN and LDA trained model using training database, the generalization capabilities of the selected model is evaluated using testing database. The working of the proposed LDA-NN-GA approach is more clearly depicted in Fig. 1.

## III. Validation Schemes and Evaluation Metrics

### A. Validation Schemes

Different validation schemes have been proposed in literature to evaluate the performance of a learning model. These schemes include k-fold cross validation, train-test holdout validation and leave one out cross validation. However, these schemes introduce subject overlap when they are utilized with data having many samples per subject. Thus, to avoid the problem of subject overlap, the most pertinent methodology is to use leave one subject out (LOSO) cross validation. Hence, we use LOSO cross validation scheme in this study. In LOSO, one subject is kept out to be tested while the model is trained on the data of all other subjects. The same process is repeated for all the subjects. Finally, the average accuracy of all subjects is calculated.

### B. Evaluation Metrics

Different evaluation metrics including accuracy, specificity, sensitivity, and Methews correlation coefficient ($MCC$) have been exploited to measure the performance of the proposed automated system. For LOSO CV, accuracy reports information about correctly classified subjects in the given dataset. Sensitivity reports information about correctly classified patients while specificity conveys information about correctly classified healthy subjects.

\begin{equation*} Accuracy = \frac
              {TP+TN}{TP+TN+FP+FN}\tag{8}\end{equation*}

where $TP$ stands for number of true positives, $FP$ for number of false positives, $TN$ for number of true negatives and $FN$ for number of false negatives.

\begin{align*}&\hspace {-20pt}Sensitivity = \frac
              {TP}{TP+FN} \tag{9}\\&\hspace {-20pt}Specificity = \frac {TN}{TN+FP}
              \tag{10}\\&\hspace {-20pt}MCC = \frac {TP \times TN - FP \times FN}{\sqrt
              {(TP+FP)(TP\!+\!FN)(TN\!+\!FP)(TN+FN)}}\tag{11}\end{align*}

$MCC$ is a more robust evaluation metric that even works for imbalanced datasets. It can have a value in the range −1 to 1.

## IV. Experimental Results and Discussion

As discussed above, the main problem with multiple types of speech data is subject overlap. Different researchers utilized different methods to avoid the problem of subject overlap. In this section, we follow the methodology used by Benba *et al.* in [1], [31]. That is we transform the multiple types of dataset into one type of datasets. After the transformation, we obtained three datasets for the training database. The first dataset contains vowel “a” sample for each subject. The second dataset contains vowel “o” sample for each subject while the third dataset contains vowel “u” sample for each subject. To evaluate performance of the proposed method, we apply LDA-NN-GA on each of the dataset in the first three experiments and in the last experiment, the generalization capabilities of the obtained model are validated by applying it on the testing database. The python code to regenerate the results can be accessed at (https://github.com/LiaqatAli007/Automated-Detection-of-Parkinson-s-Disease-Based-on-Multiple-Types-of-Sustained-Phonations-using-Lin).

### A. LOSO Cross Validation Using Vowel “u” Voice Samples of Training Database

In this experiment, only the vowel “u” voice sample for each subject are considered. After construction of the vowel “u” dataset, LOSO CV is performed using the proposed LDA based dimensionality reduction method and neural network based classification. The simulation results are reported in Table 3. From the first two rows of the table, it is clear that inappropriate network configuration gives us poor performance, thus the importance of GA for optimization of NN is validated. The proposed GA algorithm searches optimal network configuration which ensures better performance. Moreover, as genetic algorithm is population based optimization method, thus, running the proposed method for K different times will result in K different optimized models. This fact is evident from the last two rows of the table where best results *i.e*. optimal performance is achieved by two different NN architectures. In order to construct a model that would show better generalization capabilities, we will select that NN model that shows good performance on all the three types of training databases and the testing database. Moreover, it is important to note that in all the experiments, we have used population size = 15, number of generations = 10, gene mutation probability = 0.10 and for selection operator of GA we have used tournament selection with size of 3 *i.e*. three contestants.

**TABLE 3:** LOSO Cross Validation for Dataset Having Vowel “u” Voice Samples of Training Database. L: Number of Hidden Layers. $H_{1}$: Number of Neurons in the First Hidden Layer of NN. $H_{2}$: Number of Neurons in the Second Hidden Layer of NN. ACC(%) Percentage of PD Detection Accuracy. Sen.(%): Percentage of Sensitivity. Spec.(%): Percentage of Specificity

Hyperparameters			Evaluation Metrics			
$H_{1}$	$H_{2}$	L	ACC(%)	Sen.(%)	Spec.(%)	MCC
2	2	2	0.00	0.0	0.0	−1.00
2	12	2	0.00	0.0	0.0	−1.00
2	20	2	95	90	100	0.904
2	27	2	95	90	100	0.904
2	29	2	95	95	95	0.900

### B. LOSO Cross Validation Using Vowel “o” Voice Samples of Training Database

In this experiment, the vowel “o” voice sample of each subject is considered. After construction of the vowel “o” dataset, LOSO CV is performed using the proposed LDA-NN-GA method. The simulation results are reported in Table 4. The first two rows are the results of the system without using GA for optimizing NN which shows the importance of genetically optimized NN. It is important to note that the last row in Table 3 and Table 4 uses same network configuration.

**TABLE 4:** LOSO Cross Validation for Dataset Having Vowel “o” Samples of Training Database. $H_{1}$: Number of Neurons in the First Hidden Layer of NN. $H_{2}$: Number of Neurons in the Second Hidden Layer of NN. ACC(%) Percentage of PD Detection Accuracy. Sen.(%): Percentage of Sensitivity. Spec.(%): Percentage of Specificity

Hyperparameters			Evaluation Metrics			
$H_{1}$	$H_{2}$	L	ACC(%)	Sen.(%)	Spec.(%)	MCC
2	2	2	0.00	0.0	0.0	−1.00
2	15	2	87.5	85	90	0.750
2	25	2	87.5	90	85	0.750
2	20	2	92.5	90	95	0.851
2	29	2	92.5	95	90	0.851

### C. LOSO Cross Validation Using Vowel “a” Voice Samples of Training Database

In this experiment, the vowel “a” voice samples for different subjects are considered. After construction of the vowel “a” dataset, LOSO CV is performed using the proposed intelligent system. The simulation results are reported in Table 5. It is important to note that the last row in Table 3, Table 4 and Table 5 denotes the same network configuration. Hence, this network configuration offers a generalized solution to the PD detection problem using voice recordings data. The same network configuration also shows good performance on the testing database.

**TABLE 5:** LOSO Cross Validation for Dataset Having Vowel “a” Voice Samples of Training Database. $H_{1}$: Number of Neurons in the First Hidden Layer of NN. $H_{2}$: Number of Neurons in the Second Hidden Layer of NN. ACC(%) Percentage of PD Detection Accuracy. Sen.(%): Percentage of Sensitivity. Spec.(%): Percentage of Specificity

Hyperparameters			Evaluation Metrics			
$H_{1}$	$H_{2}$	L	ACC(%)	Sen.(%)	Spec.(%)	MCC
2	2	2	0.00	0.0	0.0	−1.00
2	9	2	0.00	0.0	0.0	−1.00
2	39	2	87.5	90	85	0.750
1	29	2	92.5	95	90	0.851
2	29	2	92.5	95	90	0.851

### D. LOSO Validation on Testing Database

To further validate the generalization capabilities of the constructed model based on the above experiments, in this experiment the same neural network model is tested using data of testing database. Following the approach of the previous studies, in this experiment we train our model using the data of the training database and then test it on the testing database. For validating the performance of the constructed model on the testing database, we use LOSO validation scheme *i.e*. we train model on the training database and test it on the data of each subject in the testing database. The simulation results are reported in Table 6. From the table, it can be seen that for testing database only accuracy and sensitivity has been reported and no information about specificity and MCC is given. It is due to the fact that the independent testing database is collected from PD patients only. Thus, specificity and MCC cannot be evaluated for this database.

**TABLE 6:** LOSO Validation on Testing Database. $H_{1}$: Number of Neurons in the First Hidden Layer of NN. $H_{2}$: Number of Neurons in the Second Hidden Layer of NN. ACC(%) Percentage of PD Detection Accuracy. Sen.(%): Percentage of Sensitivity

Hyperparameters			Evaluation Metrics	
$H_{1}$	$H_{2}$	$L$	ACC(%)	Sen.(%)
5	5	2	42.85	42.85
1	29	2	57.14	57.14
2	20	2	60.71	60.71
29	2	2	57.14	57.14
2	29	2	100.0	100.0
29	29	2	60.71	60.71

### E. Time Complexity Analysis of the Proposed Method

In this subsection, we evaluate the time complexity of the NN hyper-parameters optimization through GA and compare it with the conventional or baseline grid search algorithm. Additionally, the training time of the LDA based NN is much less compared to the training time of the conventional or baseline NN (trained on full features). From Table7, it is clear that the use of GA makes the optimization much faster compared to the baseline algorithm. The optimization process for both the methods *i.e*. proposed and baseline is carried out on same search space. Thus, it is proved that the proposed method has three main advantages. First, it gives outstanding performance in terms of classification accuracy. Second, the proposed method has better generalization capabilities. Third, the proposed method has lower time complexity.

**TABLE 7:** Time Complexity Analysis of the Proposed Method. Time (Sec.): Processing Time in Seconds. HPO: Hyperparameters Optimization

Method	Time (sec.)	Optimization
Proposed	9.0842	HPO
Baseline	5194.5	HPO

## V. Comparative Study

In this section, we compare the performance of our method with other methods reported for PD detection problem based on multiple types of voice recordings data. A brief description of these methods and their achieved accuracies are reported in Table 8. The comparative study is performed from two aspects *i.e*., PD detection accuracy and generalization capabilities. Usually, it is very important to check the generalization capabilities of a fitted model *i.e*. to judge that it is plausible that its predictions will carry over to fresh unseen data. Two methods are usually used for this purpose *i.e*. using a separate hold-out testing dataset and the computationally much more burdensome leave-one-out cross-validation [49]. In literature, the cross validation method is considered better than holdout method if dataset is not too large.

**TABLE 8:** Comparative Study of the Proposed Method With Previous Methods That Used All the 26 Features

Study	Method	Acc(%)	Sen.(%)	Spec.(%)
Sarkar $et~ al$. [20]	KNN + SVM	55.00 (LOSO on training database) 68.45 (LOSO on testing database)	60(Training database)	50 (Training database)
Canturk and Karabiber [29]	4 Feature Selection Methods + 6 Classifiers	57.5 (LOSO CV), 68.94 (10fold)	54.28(LOSO), 70.57(10fold)	80(LOSO), 66.92(10fold)
Eskidere $et~ al$. [50]	Random Subspace Classifier Ensemble	74.17 (10-fold CV)	Did not report	Did not report
Behroozi and Sami [51]	Multiple classifier framework	87.50 (A-MCFS)	90.00	85.00
Zhang $et~ al$. [52]	multi-edit nearest-neighbor(MENN) + ensemble learning algorithms (Random forest with MENN)	81.5 (LOSO on train database) 100 (LOSO on test database)	92.50 (Training database), 100 (Testing database)	70.50(Training database), 100(Testing database)
Benba $et~ al$. [31]	Human Factor Cepstral Coefficients (HFCC) + SVM	87.5 (LOSO on train database) 100 (LOSO on test database)	90.00 (Training database), (100.0 Testing database)	85.00 (Training database), — (Testing database)
Li $et~ al$. [30]	Hybrid feature learning + SVM	82.50 (LOSO on training database)	85.00	80.00
Vadovsk and Parali [53]	C4.5 + C5.0 + Random Forest + CART	66.5 (4-Fold CV with pronouncing numbers) 65.86 (5-Fold CV with pronouncing numbers)	Did not report	Did not report
Y. N. Zhang [54]	Stacked auto-encoders (SAE) + KELM + LSVM + MSVM + RSVM + CART + KNN + LDA + NB	94.17 (Train-Test holdout)	50.00	94.92
Benba $et~ al$. [1]	MFCC + SVM	82.5 (LOSO on train data file)	80.00	85.00
Kraipeerapun and Amornsamankul [55]	Stacking + Complementary Neural Networks(CMTNN)	Average 75 % (10-fold CV)	Did not report	Did not report
Khan $et~ al$. [56]	Evolutionary Neural Network Ensembles	90 (10-fold CV)	93.00	97.00
Cai $et~ al$. [57]	CBFO + FKNN	83.68	96.92	33.33
Proposed method	LDA-NN-GA	95% (LOSO CV on train database)	95	95
Proposed method	LDA-NN-GA	100 (LOSO CV on testing database)	100	—

In the case of PD detection based on multiple types of data, previous studies have performed two independent experiments. In the first experiment, LOSO CV on training database is performed while in the second experiment, LOSO CV on the testing database is performed. During the LOSO CV on the training database, one subject data is left out for testing purpose and the model is trained on the data of the remaining subjects present in the training database. This process is repeated until all the subjects are tested. Although, previously proposed methods constructed one generalized model for the cross validation on the training database. But, while performing LOSO CV on the testing database, they ended up with another model. For example, Benba *et al.* in [31] achieved state-of-the-art performance of 87.5% on the training database under LOSO CV using linear SVM and 100% on the testing database under LOSO CV using polynomial SVM. The generalization would have been more robust if one generalized model was constructed for both training and testing databases. In this paper, one generalized model is constructed that shows better performance on both *i.e*., training and testing databases. From literature survey depicted in Table 8, it is clear that the proposed approach achieved better results than previously proposed state-of-the-art methods.

## VI. Limitations of the Study

Although, the proposed LDA-GA-NN learning system outperformed the previously proposed methods by utilizing same original features set that was utilized in the previous studies. However, there are some limitations in the multiple types of phonations dataset that have been widely adopted for PD detection problem. The first limitation is in the independent dataset (testing dataset) which was only collected from PD patients and is highly imbalanced *i.e*., the healthy class has no representation in the testing database. Additionally, no information about UPDRS is provided for the subjects of the testing database. The second limitation is missing information about the feature extraction process *e.g*., was the extraction of features corrected for pitch halving/doubling? Third, information about speech severity and whether the PD patients were investigated in the OFF or ON state, are also missing.

Another limitation is the imbalanced gender in the dataset. Hence, the results may be biased. In order to obtain unbiased results, we develop another experimental setting in which we removed gender-dependent features from the feature space *i.e*. jitter (local), shimmer (local), all 4 pulses/periods measures, and all 5 pitch measures have been removed and the remaining features have been considered for the new experiments. It was observed that the maximum accuracy of 80% was obtained under LOSO cross validation on training database and 82.14% on testing database for vowel “o” dataset using two types of neural network architectures. The first neural network has two hidden layers with $H_{1}=6$ and $H_{2}=7$ while the second neural network has $H_{1}=1$ and $H_{2}=7$. Similarly, for vowel “a” dataset, we obtained 85% of accuracy on training database and 64.28% of accuracy on testing database by utilizing a neural network with two hidden layers and $H_{1}=32$ and $H_{2}=2$. This degradation in accuracy clearly highlights the limitations in the multiple types of phonation dataset which have been widely used for PD detection problem. And similar accuracy degradation is also expected in the previous methods reported in Table 8 if the gender dependant features are eliminated from the feature space.

## VII. Conclusion

In this paper, we developed a hybrid intelligent system for PD detection based on multiple types of sustained phonations data. The developed system uses LDA model for dimensionality reduction and neural network model for classification. The architecture of the neural network model was optimized using genetic algorithm. Experimental results showed that the developed intelligent system was capable of discriminating between PD patients and healthy subjects with an accuracy of 95% on training database and 100% on testing database using all the collected features of the dataset. However, the limitation of gender imbalance in the dataset was highlighted and hence the gender dependent features were eliminated and the proposed system was again simulated. Consequently, we obtained 80% accuracy on training database and 82.14% on testing database. The results on both *i.e*. training and testing databases were obtained using one generalized model which also has the benefit of lower complexity. Thus, based on the experimental results, it can be concluded that the proposed automated system has the potential to help physicians improve the quality of decision making during diagnosis process of PD patients.
